# The effect of light curing intensity on bulk-fill composite resins: heat generation and chemomechanical properties

**DOI:** 10.1080/26415275.2021.1979981

**Published:** 2021-09-29

**Authors:** Wendy Jingwen Wang, Anastasiia Grymak, John Neil Waddell, Joanne Jung Eun Choi

**Affiliations:** Sir John Walsh Research Institute, Faculty of Dentistry, University of Otago, Dunedin, New Zealand

**Keywords:** Resin composite, bulk-fill, polymerisation, heat generation, light curing, hardness, degree of conversion

## Abstract

**Objectives:**

The aim of this study was to assess the effect of light curing intensity and wavelength spectrum on heat generation and chemomechanical properties of bulk-fill composites.

**Methods:**

Four bulk-fill restorative materials (Filtek bulk-fill, Tetric PowerFill bulk-fill, Beautifil Bulk restorative and Admira Fusion X-tra were used in this study. A total of 100 cylindrical specimens of each composite (*n* = 25/group) were prepared, then cured using monowave light curing unit (LCU) with a single light intensity of 1470 mW/cm^2^, and polywave LCU with three different light intensities (1200,2100, 3050mW/cm^2^). The temperature change during polymerisation was measured by five K-type thermocouples placed in each 1 mm layer from top to bottom. Hardness and degree of conversion of composites at each level were evaluated. Results were statistically analysed.

**Results:**

The use of polywave LCU resulted in statistically higher peak temperatures ranging between 31.4–63.5 °C compared to the temperature generated by monowave LCU ranging between 29.5–60 °C (*p* < .05). Curing using polywave LCU with the highest light intensity of 3050 mW/cm^2^ caused the highest peak temperature irrespective of the composite types. There was no significant difference in hardness with different light curing intensities and curing times, regardless of the bulk-fill resin materials (*p* > .05). A positive correlation was also found between the hardness and the DoC of the four bulk-fill composites.

**Conclusion:**

The change in temperature during polymerisation of bulk-fill composites were found to be proportional to the increase in light curing intensity. Mechanical properties of the bulk-fill composites were dependent on the composition and the type of photoinitiators.

## 1. Introduction

It is well established that successful composite dental restorations require adequate mechanical properties against the consistent masticatory forces and erosive oral environment [1]. One major factor that affects these properties is the degree of conversion (DoC) through photo-activation *via* light curing [1,2]. Bulk-filled composite resins (BCR) are becoming popular in restorative dentistry as they enable bulk placement of dental fillings in thick increments (rather than multi-layer applications for conventional composites) while reducing clinical time and cost. BCRs also have a high degree of conversion, contributing to improved mechanical properties such as hardness as well as improved long-term clinical durability [3–5]. When any composite restoration experiences a low degree of resin polymerisation and inadequate depth of cure, it can have a decreased surface hardness, making the final restoration more prone to deformation and dental wear, increasing the risk of restoration failure such as secondary caries [5].

In order to ensure efficient polymerisation of thick layers, dentists tend to cure BCRs with higher intensity light. The current literature suggests that there is an association between light intensity, degree of conversion and surface hardness [1,3]. High light intensity (radiance emittance) at effective wavelength provides sufficient energy to activate initiators, therefore enabling adequate resin polymerisation and good mechanical properties [6]. A recent study conducted by Par et al. [4] have found that significantly higher microhardness was obtained with a higher intensity curing unit. These recent studies all suggest that there is a positive association where the mechanical strength is directly related to increased light curing intensity. However, some studies reported conflicting results. For example, Shimokawa et al. [5], considered light curing units with high intensity and reported that it can lead to increased polymerization stress as a result of faster polymerization rates. High intensity light also produces more radicals very rapidly resulting in early bi-radical termination which lowers the degree of conversion of the BCRs [7].

Currently, many manufacturers attempt to improve the properties of light-emitting diode (LED) light curing units (LCU). One feature introduced recently is the broad-spectrum polywave technology. This is added to enhance photoactivation and the associated degree of polymerisation, especially in BCRs that incorporate co-initiators which require lower wavelength to activate the polymerisation process [8–10]. However, recent research suggested that multiple wavelength outputs resulted in an increase in the inhomogeneity of irradiance leading to uneven cure of the composite and reduction of overall mechanical properties [8,11,12].

There is also a proposed relationship between light curing intensity and heat generation [13]. However, there are currently no studies reporting the effect of heat generation related to curing light intensity on the hardness of the BCRs. There is also a gap in the literature reporting on the most effective curing light intensity that gives the maximum mechanical properties to the BCRs, while producing the least amount of heat to protect the pulp. Scientific evidence on this will let clinicians know the most effective way to cure BCRs to provide dental fillings with maximum mechanical properties to the patients while reducing the clinical time and costs.

Therefore, the purpose of our study was to investigate the effect of light curing intensity and wavelength spectrum on heat generation of four brands of BCRs and their influence on the hardness and degree of cure, when cured with two different types of LCUs (monowave and polywave). The null hypotheses were:

1. There are no significant differences in temperature and mechanical properties amongst various commercial BCR materials.
2. There are no significant differences in temperature change and mechanical properties amongst various commercial BCR materials when cured by a monowave LCU compared with curing by polywave LCU.
3. There are no correlations between the temperature increase and the microhardness of BCR materials.

## 2. Materials and methods

### 2.1. Specimen preparation and curing

**Figure 1.**
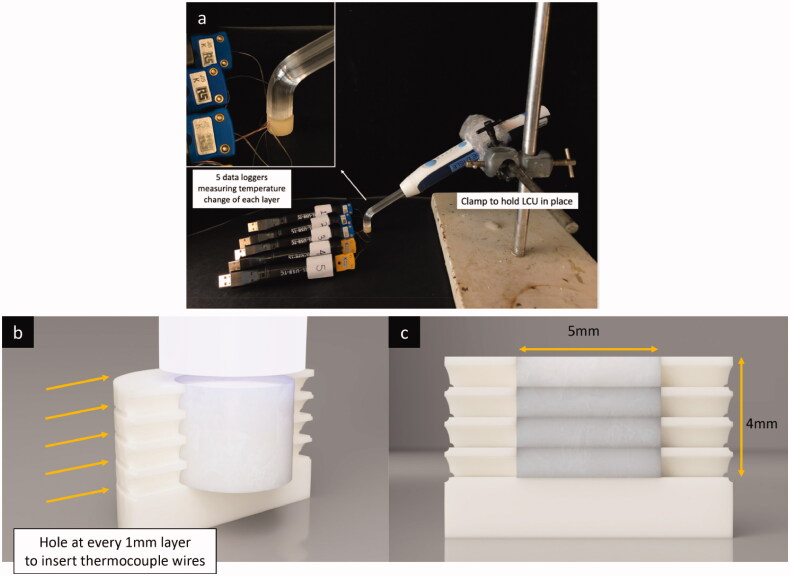
(a) Photograph showing the experimental setup for measurement of the heat generated by bulk-fill composite resins during photoactivation. A clamp was used to hold the light curing unit (LCU) in place and five data loggers connected to thermocouples were used to measure temperature change of each layer; (b and c) Diagram showing the design and dimension of the 3 D printed resin mould.

Four commercially available bulk-fill composite resins; Filtek One bulk-fill resin (3M), Tetric PowerFill bulk-fill (Ivoclar Vivodent), Beautifil-Bulk restorative (Shofu) and Admira Fusion X-tra (Voco) were used in this study as presented in Table 1. A sample size calculation was performed by referring to previous experiments of similar nature and outcomes using the software G*power v3.0.10 (Heinrich-Heine-Universität Düsseldorf). The calculation showed that 25 specimens per group are required. Hence, a total of 25 cylindrical specimens of each BCR (total, *n* = 100) were prepared for the study. As shown in Figure 1, moulds were created using tooth-coloured resin (Freeprint Temp 3D print resin, Shade A2) through 3D printing (Asiga Max, Asiga) which simulate the dimension of a normal molar tooth and the space created for a bulk-fill restoration at the centre. Small cavities were created at the side of the mould at each millimetre to allow the placement of thermocouples (Figure 1(a,b)).

**Table 1.** Composition of the materials tested as provided by the manufacturer.

Materials	Manufacturer increment thickness (mm)	Matrix	Filler	Filler % (wt)
Beautiful Bulk Flow GIOMER (Shofu Dental Cooperation)	4	Bis-GMA UDMA Bis-MPEPP TEGDMA	S-PRG filler based on F-Br-Al-Si-glass	72.5
Tetric PowerFill (Ivoclar Vivadent)	4	Bis-GMA Bis-EMA UDMA Aromatic dimetheacrylate DCP	barium glass, ytterbium trifluoride, mixed oxide and copolymers	79–80
Admira Fusion X-tra (Voco GmbH)	4	Ormocer (organically modified ceramics)	Ba-Al-Si-glass/silica nanoparticles	84
Filtek® One Bulk Fill (3M ESPE)	5	AFM AUDMA UDMA 1, 12-docecane-DMA	Non-agglomerated 20nm silica Non-agglomerated 4–11nm zirconia Aggregated zirconia/silica cluster filler Ytterbium trifluoride filler	76.5

The BCRs were injected and packed in 1 mm increments after placement of thermocouples on top of each increment. The final BCR increment was then immediately light cured 1 mm away from the restorative surface at once using the monowave LCU (Elipar DeepCure-L LED Curing Light, 3 M; a single light intensity of 1470 mW/cm^2^ with a curing time of 20 s) and a polywave LCU (Bluephase PowerCure, Ivoclar Vivadent, Liechtenstein, providing three different light intensities 1200, 2100, 3050 mW/cm^2^ with curing times of 3, 5, 10 and 20 s, respectively. The LCUs were held into position using a clamp to enable consistent distribution of light during the polymerisation process (Figure 1(a)). The application of composite into the mould and curing with LCU was handled by a single blinded operator for consistent data collection. Light intensity (radian emittance) was measured (*n* = 10 per group/curing modes) using a dental radiometer (Bluephase meter III; Ivoclar Viva-dent, Schaan, Liechtenstein) as a confirmation of the intensity/emittance claimed by the manufacturer.

### 2.2. Temperature measurement

A total of five (K-type) thermocouples were connected and subsequently used in the experiment to measure real-time temperature change. Four (K-type) thermocouples were inserted manually though the cavity into the centre of the mould before placing a 1 mm layer of BCR on top (Figure 1). The BCR was then packed into the mould in 1 mm increment using flat plastic instrument and condensed. One (K-type) thermocouple was placed manually on the top surface at the centre after packing the restorative material. This setup enables the measurement of real-time temperature change at each millimeter using a data logger (GFX Data Logger Series and EL-USB-TC, Lascar Electronics Inc, USA) (Figure 1(a)). Each data logger was being pre-setup to start at the same time accurate to millisecond to enable simultaneous temperature measurement from each layer. The data loggers started to record real-time temperature change at the beginning of the light curing and stopped after two minutes of cooling time. The starting time was recorded in milliseconds and the finishing time was calculated accurate to milliseconds using the formula: starting time + curing time + 2 s.

This procedure was repeated five times for each group, after which the measured data was converted and transferred into Microsoft Excel using EasyLog USB software and the mean and standard deviation were calculated.

### 2.3. Evaluation of hardness

**Figure 2.**
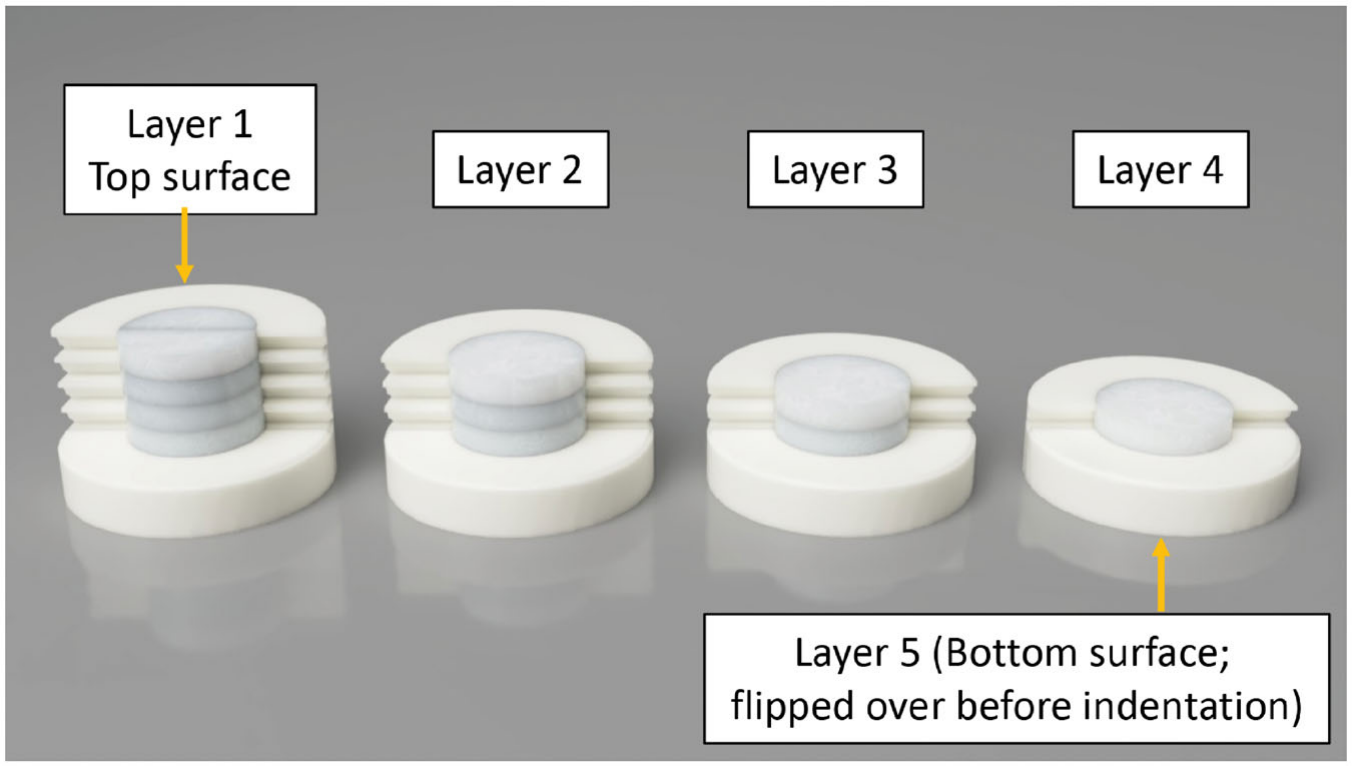
Diagram showing the specimen prepared for nanoindentation and FTIR analysis; The specimen was ground down to different thicknesses.

After temperature measurement, specimens were polished down to measure the hardness and elastic modulus of 4 mm (top surface), 3 mm, 2 mm, 1 mm and the bottom surface (Figure 2). The polishing was done with TegraPol-21 (Struers, Germany) polishing machine with sand papers of 120, 500, 1200 and 2400 grits to achieve a polished surface suitable for nanoindentation. Nano-indentation was performed at room temperature, 22 °C, in a UMIS nano-indentation system (UMIS) at a static load of 60 mN with 20 indents per specimen across the surface. Compliance of the load frame for the nano-indentation unit was 0.2 nm/mN. Post-data analyses of elastic modulus and hardness were performed using IBIS 2 software (Fischer-Cripps Laboratories).

### 2.4. Evaluation of degree of conversion

To measure the degree of conversion (DoC), an ATR-FTIR (Brukers) operating from 400 to 4000 cm-1 was utilised. The FTIR spectra of uncured and cured composites (of each layer level) were analysed for diffuse absorption. The measurement was recorded as absorbance values. The number of double-carbon bonds that are converted into single bonds provides the DoC of the BCRs. The percentage of unreactive carbon-carbon double bonds (% C=C) was determined from the ratio of the absorbance intensity of aliphatic C=C (peak 1635 cm^−1^) to that of aromatic C–C (peak at 1608 cm^−1^). The DoC was determined according to the following equation:

$$\mathrm{DoC}\;(\%)\;=(1-\frac{\frac{\mathrm{Aliphatic}}{\mathrm{Aromatic}}\;\mathrm{area}\;\mathrm{cured}\;\mathrm{material}}{\frac{\mathrm{Aliphatic}}{\mathrm{Aromatic}}\;\mathrm{area}\;\mathrm{uncured}\;\mathrm{material}})\;\times 100$$

### 2.5. Statistical analysis

The collected temperature data were expressed as mean and standard deviation (SD). The effects of high and low curing light intensities on the mechanical properties of the tested composites were statistically analysed using one-way analysis of variance (ANOVA) and Bonferroni post-hoc analysis, using SPSS software (Version 27, IBM). All tests were performed at a significance level of *p* < .05. A spearman correlation coefficient analysis was also conducted to determine the correlation between the microhardness and DoC.

## 3. Results

### 3.1. Characterisation of the light curing units and temperature change in BCRs

Firstly, the actual polymerisation light curing intensity (mW/cm^2^, radiant emittance) of each LCUs and each curing modes used in the current study was analysed and compared with the manufacturers’ claim. The actual mean light intensity recorded for both the monowave and the polywave LCUs were lower than what has been reported by the manufactures (Table 2). The polywave LCU had a higher standard deviation in the light intensity measured by the radiometer, compared to that of the monowave LCU.

**Table 2.** Light intensity characterisation (mW/cm^2^) of light curing units (LCUs) used depending on the curing modes, measued by a radiometer (*n* = 10).

	Monowave 20s	Polywave 3s	Polywave 5s	Polywave 10s	Polywave 20s
Light curing intensity (mW/cm^2^) claimed by manufacturers	1470	3050	2100	1200	1200
Actual mean light intensity (mW/cm^2^)	874 ± 6.9	2546 ± 40.8	1746 ± 53.3	974 ± 12.6	963 ± 11.6

The temperature profile including the peak temperature increases during polymerisation with different LCU types (monowave vs. polywave), light intensity and BCR materials, are shown in Table 3. The result using the monowave LCU (Elipa DeepCure-L) showed that the peak (maximum) temperature during polymerisation, which included both the temperature rise due to irradiance from the curing light and the exotherm from the reaction, ranged between 29.5–60.0 °C. The highest peak temperature (60 °C) was from the top layer of the Tetric PowerFill BCR material (Figure 3), while bottom layers, especially from Beautifil BCR, showed the lowest peak temperature of 29.5 °C. The peak temperature increases with the application of the polywave LCU (Bluephase PowerCure, Ivoclar vivadent) was ranged between 31.4–63.5 °C. The highest peak temperature (63.5 °C) was from the top layer of the Tetric PowerFill BCR at the highest light intensity of 3050 mW/cm^2^. The bottom layer Beautifil BCR showed the lowest peak temperature of 31.4 °C when polymerised under the lowest light intensity of 1200 mW/cm^2^.

Figure 3.Temperature profiles of the four BCRs (a) Filtek; (b) PowerFill; (c) Admira; (d) Beautifill.

**Figure:**
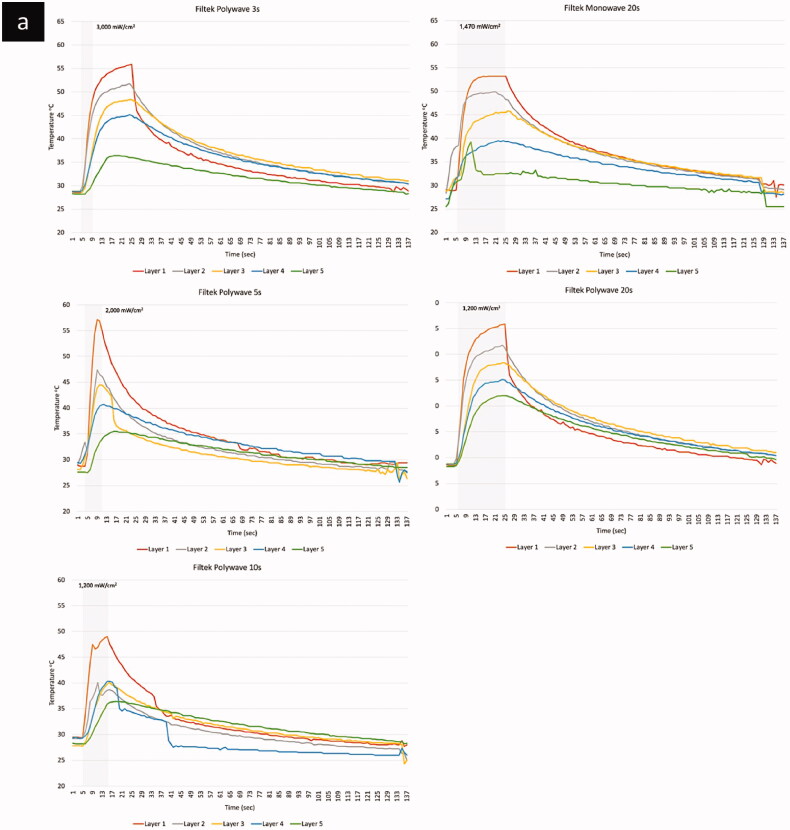

**Figure:**
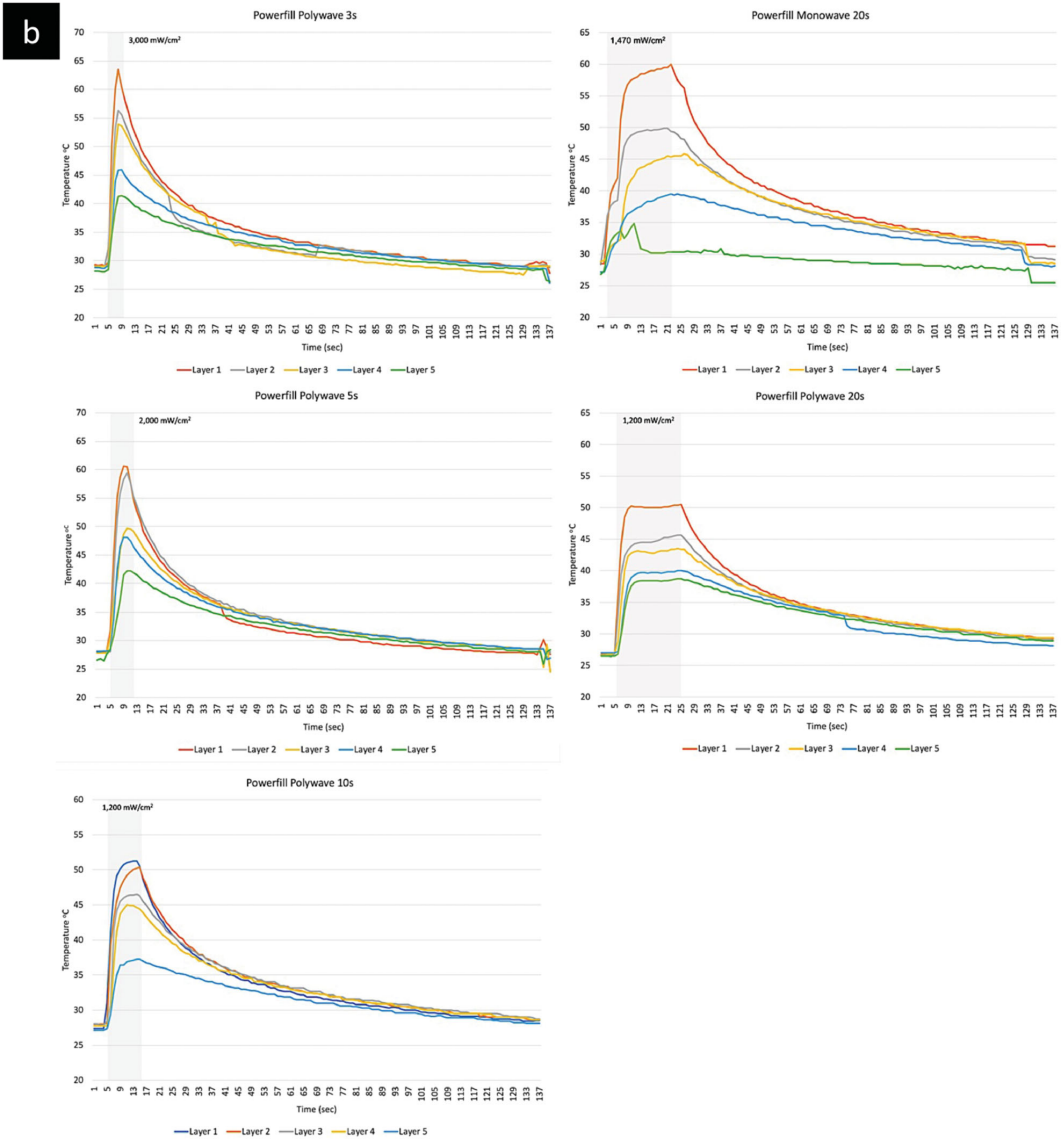
(Continued).

**Figure:**
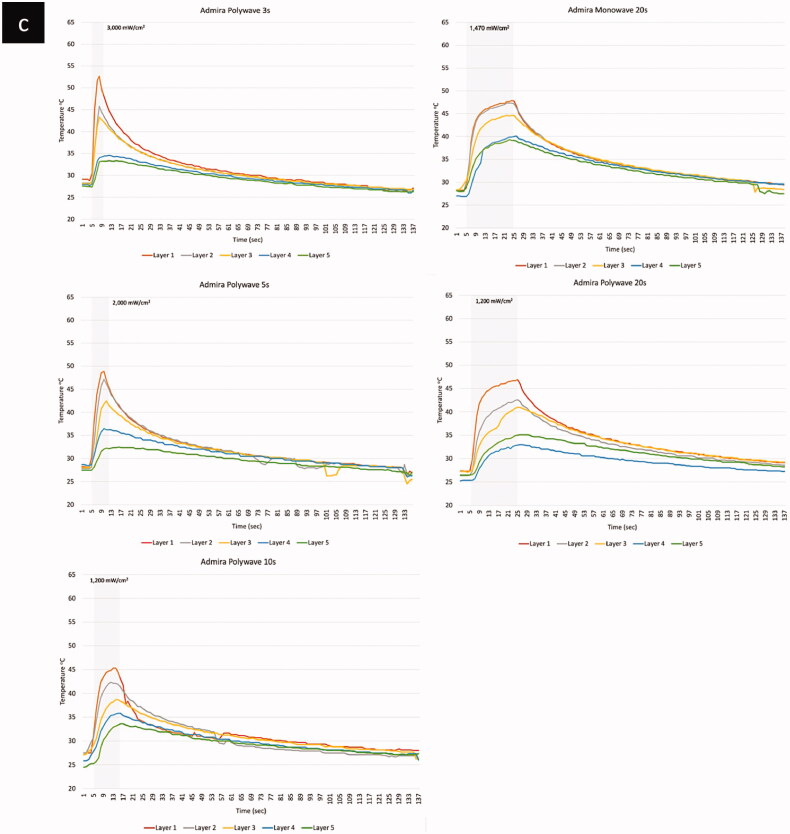
(Continued).

**Figure:**
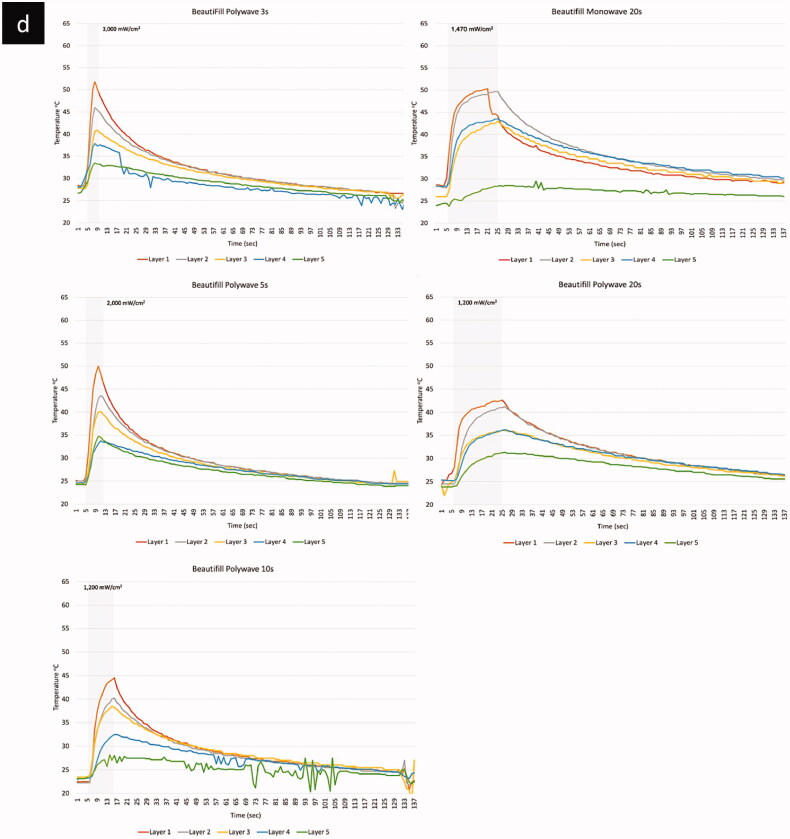
(Continued).

**Table 3.** Temperature data measured at each layer in all four BCRs cured by a monowave and a polywave light curing unit at in various light intensities and corresponding curing time.

Light curing units	Curing time (seconds)		Temperature ^o^C											
			Filtek One Bulk-fill			Beautifil Bulk Restorative			Admira Fusion X-tra			Tetric PowerFill		
			Mean (± S.D)	Max (Peak)	Min	Mean (± S.D)	Max (Peak)	Min	Mean (± S.D)	Max (Peak)	Min	Mean (± S.D)	Max (Peak)	Min
Monowave	20s	Layer 1 (Top)	38.1 (± 7.3)	53.2	27.5	34.6 (± 6.1)	50.3	28.5	35.3 (± 5.7)	47.9	28.3	39.3 (± 8.9)	60.0	28.5
		Layer 2	37.4 (± 6.2)	49.9	28.4	36.4 (± 6.3)	49.7	28.0	35.3 (± 5.6)	47.3	28.0	37.4 (± 6.2)	49.9	28.4
		Layer 3	36.5 (± 5.0)	45.8	28.5	34.0 (± 4.3)	43.0	26.0	34.8 (± 4.9)	44.6	27.9	36.4 (± 5.0)	45.8	28.5
		Layer 4	34.0 (± 3.3)	39.5	27.2	35.2 (± 4.3)	43.5	28.3	33.5 (± 3.4)	40.1	26.9	34.0 (± 3.3)	39.5	27.2
		Layer 5 (Bottom)	30.4 (± 2.3)	39.3	25.5	27.0 (± 1.0)	29.5	23.8	33.0 (± 3.4)	39.3	27.5	29.0 (± 1.7)	34.8	25.5
Polywave	3s	Layer 1 (Top)	34.6 (± 7.7)	55.9	25.8	32.0 (± 5.7)	51.8	26.7	31.7 (± 5.4)	52.7	26.5	34.4 (± 7.4)	63.5	21.9
		Layer 2	35.5 (± 7.1)	51.8	25.0	31.5 (± 4.9)	46.0	23.3	30.9 (± 4.2)	45.8	26.7	33.1 (± 6.4)	56.3	23.2
		Layer 3	35.5 (± 6.1)	48.3	27.2	30.7 (± 3.8)	40.9	24.8	30.9 (± 3.9)	43.3	26.8	32.5 (± 6.5)	53.9	23.6
		Layer 4	34.3 (± 5.3)	45.1	26.1	28.5 (± 3.3)	37.9	23.0	29.7 (± 2.4)	34.6	26.0	32.6 (± 4.6)	45.9	24.0
		Layer 5 (Bottom)	30.7 (± 3.1)	36.4	35.5	28.7 (± 2.3)	33.5	24.5	29.2 (± 2.2)	33.4	26.3	31.6 (± 3.7)	41.4	24.9
	5s	Layer 1 (Top)	34.4 (± 6.6)	57.1	28.1	29.5 (± 5.8)	50.0	24.4	32.3 (± 4.8)	48.9	26.3	33.0 (± 7.6)	60.6	21.0
		Layer 2	32.3 (± 4.5)	47.4	27.8	29.0 (± 4.7)	43.6	24.5	31.9 (± 4.7)	47.2	26.3	33.8 (± 7.4)	54.9	22.0
		Layer 3	31.3 (± 4.0)	44.5	25.5	28.4 (± 4.1)	40.1	24.4	31.5 (± 3.9)	42.5	24.5	32.9 (± 5.9)	49.7	19.2
		Layer 4	33.2 (± 3.5)	40.7	25.7	27.6 (± 2.7)	33.8	24.4	30.9 (± 2.5)	36.5	26.0	32.7 (± 5.3)	48.1	21.4
		Layer 5 (Bottom)	31.4 (± 2.3)	35.5	27.5	27.0 (± 2.8)	34.8	23.9	29.6 (± 1.7)	32.5	26.4	31.6 (± 4.1)	42.2	23.6
	10s	Layer 1 (Top)	32.3 (± 5.7)	49.0	26.5	29.0 (± 5.3)	44.5	20.8	31.5 (± 4.2)	45.3	27.5	33.4 (± 6.2)	51.3	25.4
		Layer 2	30.2 (± 3.5)	40.1	24.6	28.4 (± 4.4)	40.3	21.8	30.9 (± 4.5)	42.4	26.0	33.6 (± 5.9)	50.4	25.3
		Layer 3	31.1 (± 3.7)	40.1	23.7	28.6 (± 4.0)	38.5	18.3	31.1 (± 3.1)	38.7	26.2	33.4 (± 5.2)	46.5	24.1
		Layer 4	28.6 (± 3.8)	40.3	25.0	27.1 (± 2.5)	32.5	23.0	30.0 (± 2.5)	35.8	25.8	32.9 (± 4.7)	45.0	24.9
		Layer 5 (Bottom)	31.4 (± 2.7)	36.4	26.4	25.1 (± 1.7)	28.2	20.3	29.3 (± 2.1)	33.6	24.8	31.1 (± 2.9)	37.3	24.4
	20s	Layer 1 (Top)	34.6 (± 7.7)	55.9	25.8	32.3 (± 5.1)	42.7	24.3	34.8 (± 5.5)	46.9	27.3	35.0 (± 7.1)	50.5	23.8
		Layer 2	35.5 (± 7.1)	51.8	25.0	31.6 (± 4.7)	41.3	24.5	33.3 (± 4.3)	42.6	26.4	33.9 (± 5.6)	45.6	24.8
		Layer 3	35.5 (± 6.1)	48.3	27.2	30.3 (± 3.3)	36.1	22.0	33.4 (± 3.7)	41.0	27.1	33.7 (± 5.0)	43.5	25.1
		Layer 4	34.3 (± 5.3)	45.1	26.1	30.5 (± 3.1)	36.3	25.3	29.5 (± 1.9)	33.0	25.3	32.1 (± 4.5)	40.0	23.2
		Layer 5 (Bottom)	33.4 (± 4.5)	42.0	26.4	28.2 (± 2.0)	31.4	23.9	31.2 (± 2.3)	35.1	26.5	32.3 (± 3.7)	38.7	25.7

**Figure 4.**
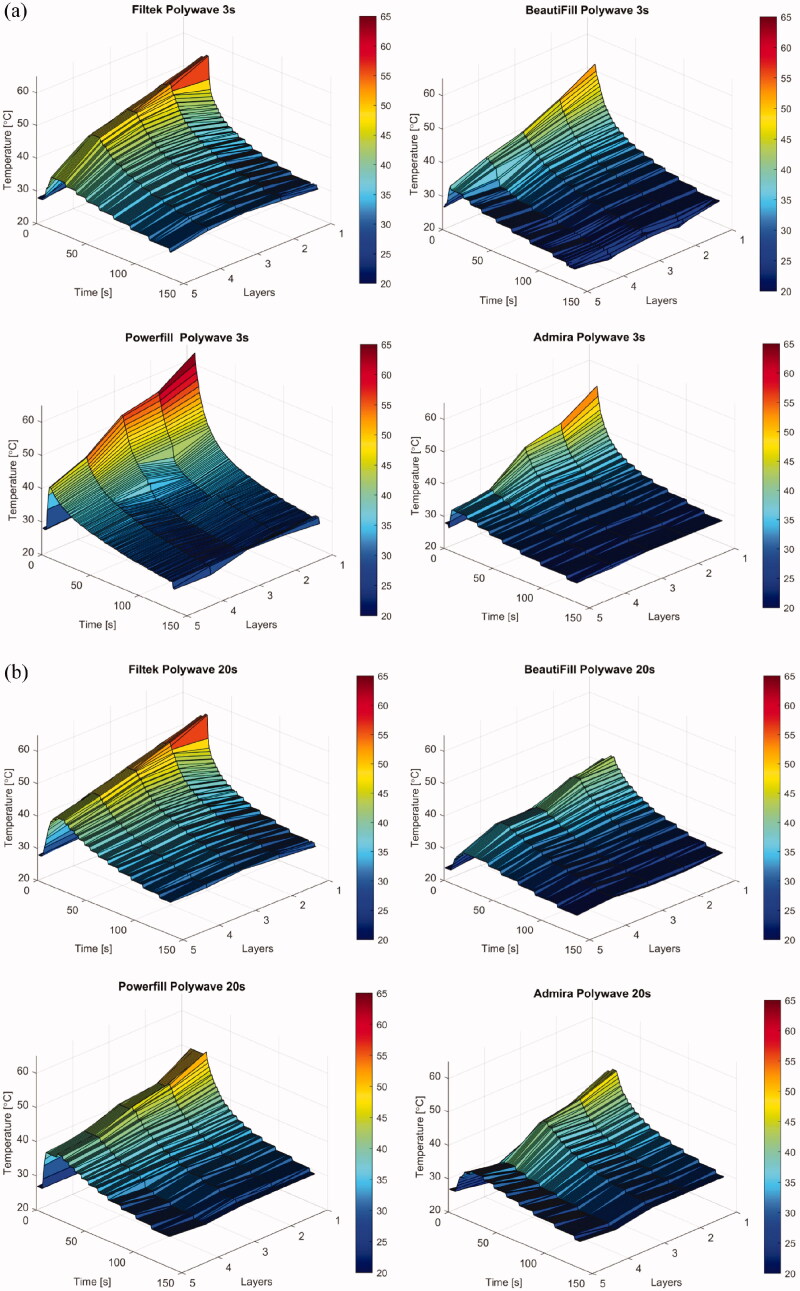
3-dimensional temperature profiles of the four BCRs showing the comparisons when cured with different LCUs and light curing intensities (a) by polywave 3 s; (b) polywave 20 s.

The light curing unit types and light intensity significantly affected the measured temperature increases and pattern of each composite (*p* < .01). There was a significant difference in temperature measurements between the monowave and polywave LCUs with different light intensity and corresponding curing time (*p = .009*) (Figure 4). However, when the same amount of curing time was applied with/without the same LCU type, no significant changes were found despite the difference in light curing intensity. The line graphs presented in Figure 2 have shown that the light curing intensity, its corresponding curing time as well as LCU type tend to have an effect of the pattern of temperature increase during light curing and the subsequent polymerisation reaction in different BCR materials. When light cured for 20 s, irrespective of the LCU type and modes, a similar pattern was shown in Filtek One and Tetric PowerFill BCR, there was a sharp increase in temperature to its peak, then it reaches plateau, which retains at the peak temperature until the end of light curing period. However, Beautifil and Admira Fusion X-tra BCR material showed a different pattern when cured for 20 s with the same light curing intensity and curing time. The temperature increase was in two stages: a sharp temperature increases in the first 5 s, after which the temperature increasing rate slowed down with a more graduate increase to peak temperature without reaching a plateau. When cured using the polywave LCU with higher light intensity and corresponding shorter curing time (3 s, 5 s, 10 s), a sharper temperature increase was resulted until it reached the peak temperature. These patterns can also be seen in the 3-dimension temperature profile graphs shown in Figure 4.

The temperature profile during the two minutes cooling down period showed slight difference in pattern between different restorative materials when cured by the different LCUs (monowave or polywave). The BCRs cured by the polywave LCU in general, showed a gradual decrease in temperature with minimal temperature difference between layers.

### 3.2. Microhardness

**Figure 5.**
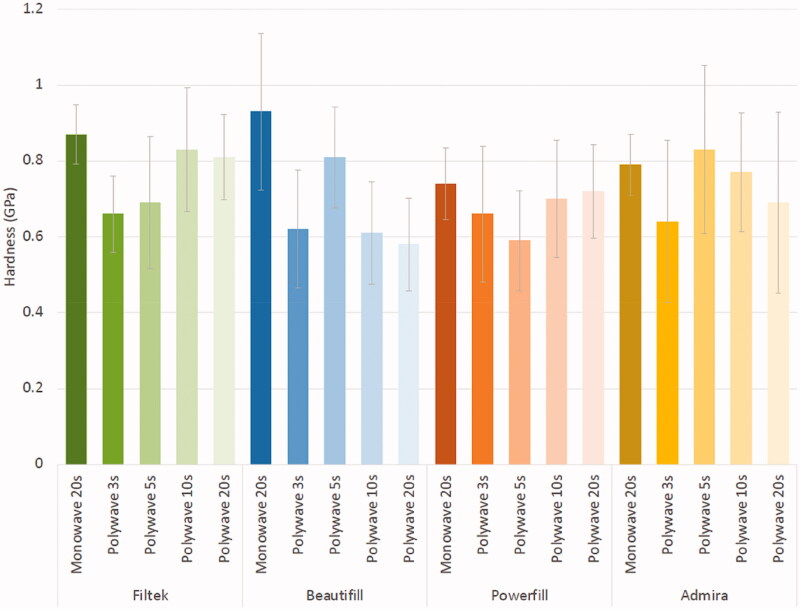
Graph showing the mean (standard deviation) hardness of four BCRs studied.

The total sample showed that the use of the monowave LCU resulted in higher microhardness values (0.50–1.03 GPa) than did the polywave LCU (0.30 − 0.99 GPa) (Table 4). As shown in Figure 5, the application of the polywave LCU has resulted in a general skew-to-the-left pattern regardless of the curing light intensity for overall hardness of the BCRs. While the layer that had the highest hardness differed for each BCR material, the top restorative surface tended to have the lowest hardness in all four materials (Table 4). Statistical analysis also revealed that in general, regardless of the light intensity and type of LCU used, the top layer of the BCR materials showed significantly lower microhardness compared to the other layers (*p* < .05). Furthermore, there was also no significant difference in hardness with different light curing intensities and curing times regardless of the BCR materials (*p* > .05).

**Table 4.** Hardness (GPa) measured at each layer in all four BCRs cured by a monowave and a polywave light curing unit at in various light intensities and corresponding curing times.

Microhardness (GPa)	BCR	Layer 1	Layer 2	Layer 3	Layer 4	Layer 5
Monowave LCU	Filtek	0.93 ± 0.38	0.75 ± 0.33	0.83 ± 0.29	0.94 ± 0.29	0.88 ± 0.28
	Beautifill	0.94 ± 0.47	1.03 ± 0.27	1.00 ± 0.32	0.78 ± 0.28	0.89 ± 0.05
	PowerFill	0.64 ± 0.23	0.88 ± 0.36	0.75 ± 0.50	0.93 ± 0.43	0.50 ± 0.01
	Admira	0.75 ± 0.41	0.94 ± 0.34	0.56 ± 0.20	0.96 ± 0.24	0.76 ± 0.23
Polywave LCU 3s	Filtek	0.48 ± 0.16	0.79 ± 0.09	0.68 ± 0.07	0.69 ± 0.10	0.65 ± 0.30
	Beautifill	0.40 ± 0.07	0.47 ± 0.14	0.68 ± 0.27	0.93 ± 0.14	0.61 ± 0.15
	PowerFill	0.42 ± 0.07	0.74 ± 0.22	0.67 ± 0.11	0.84 ± 0.22	0.64 ± 0.11
	Admira	0.55 ± 0.25	0.81 ± 0.14	0.47 ± 0.09	0.67 ± 0.18	0.70 ± 0.20
Polywave LCU 5s	Filtek	0.55 ± 0.25	0.72 ± 0.22	0.85 ± 0.25	0.78 ± 0.28	0.55 ± 0.10
	Beautifill	0.60 ± 0.21	0.89 ± 0.20	0.87 ± 0.24	0.88 ± 0.21	0.79 ± 0.35
	PowerFill	0.43 ± 0.14	0.64 ± 0.21	0.67 ± 0.13	0.63 ± 0.17	0.60 ± 0.12
	Admira	0.53 ± 0.21	0.94 ± 0.39	0.80 ± 0.28	0.90 ± 0.40	0.97 ± 0.22
Polywave LCU 10s	Filtek	0.65 ± 0.25	0.74 ± 0.24	0.84 ± 0.24	0.95 ± 0.25	0.95 ± 0.16
	Beautifill	0.42 ± 0.20	0.50 ± 0.17	0.81 ± 0.32	0.65 ± 0.32	0.68 ± 0.20
	PowerFill	0.60 ± 0.17	0.69 ± 0.13	0.64 ± 0.15	0.91 ± 0.19	0.65 ± 0.14
	Admira	0.88 ± 0.26	0.75 ± 0.09	0.79 ± 0.22	0.79 ± 0.3	0.66 ± 0.19
Polywave LCU 20s	Filtek	0.46 ± 0.11	0.77 ± 0.19	0.99 ± 0.2	0.97 ± 0.4	0.85 ± 0.21
	Beautifill	0.40 ± 0.13	0.30 ± 0.10	0.67 ± 0.11	0.80 ± 0.27	0.75 ± 0.2
	PowerFill	0.47 ± 0.09	0.89 ± 0.32	0.79 ± 0.2	0.74 ± 0.19	0.69 ± 0.1
	Admira	0.5 ± 0.28	0.47 ± 0.22	0.6 ± 0.17	0.97 ± 0.32	0.93 ± 0.41

No correlations were found between the light curing intensity and microhardness of the BCR materials (Figure 5). The highest hardness obtained from Admira Fusion X-tra (0.83 GPa) and Beautifil BCR material (0.81 GPa) were obtained by curing with the light intensity of 2000 mW/cm^2^ for five seconds. However, this curing light intensity resulted in the lowest hardness value (0.60 GPa) in Tetric PowerFill BCR, which obtained its highest hardness (0.72 GPa) from curing with the light intensity of 1200 mW/cm^2^ for 20 s.

### 3.3. Degree of conversion

**Figure 6.**
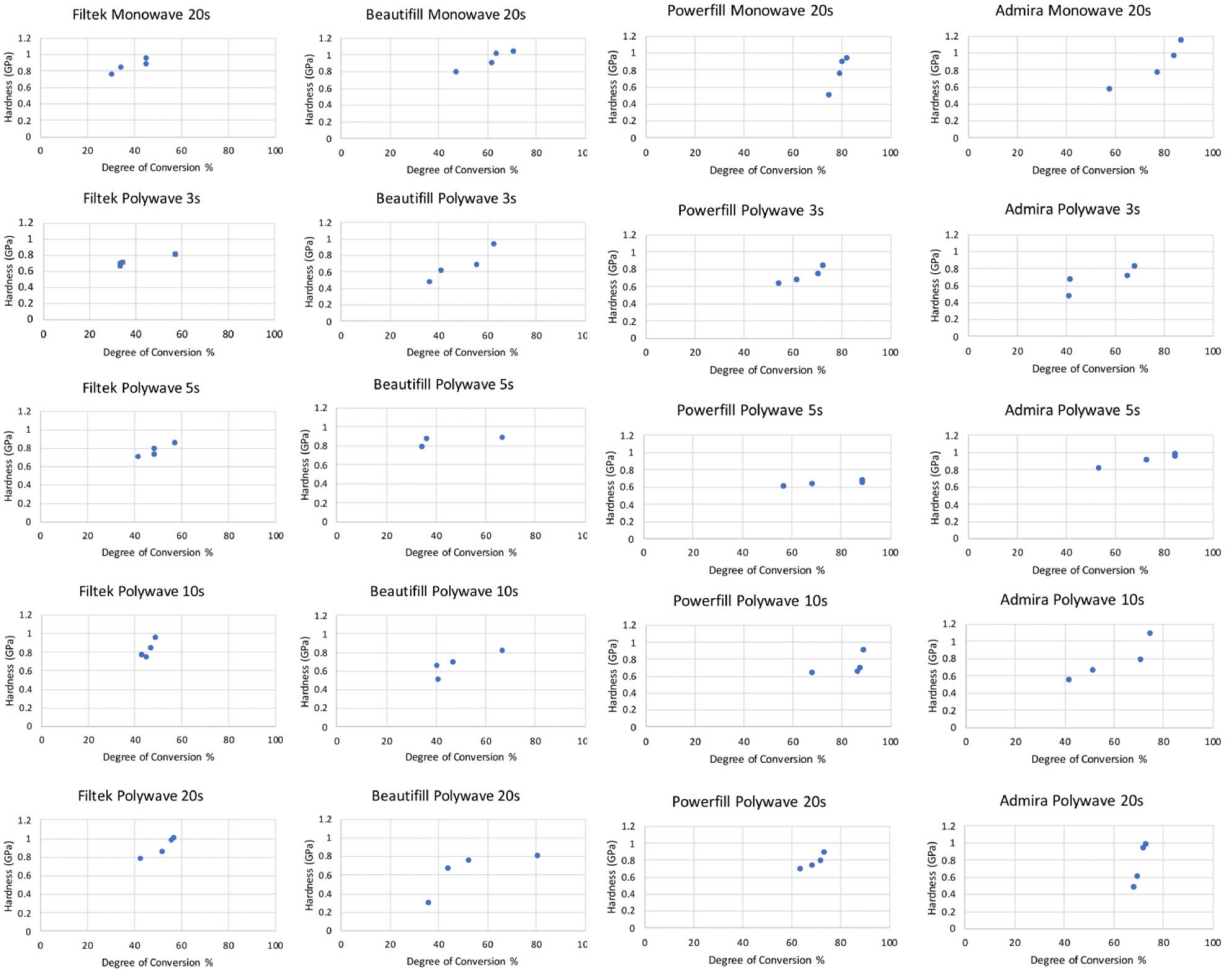
Graphs showing the correlation between the hardness and degree of conversion of the four BCRs when cured with different LCUs and light curing durations.

The degree of conversion (DoC) of different layers of the four BCR materials when cured by LCUs at different intensities are shown in Table 5. Tetric PowerFill BCR showed the highest DoC (89.21%). The statistical analysis revealed that the DoC of Tetric PowerFill was significantly higher than the DoC of Beautifil and Filtek One BCR regardless of the surface layer (*p* < .05). Furthermore, Filtek One bulk-fill restorative material showed the lowest DoC (30.62%), which was significantly lower compared with Tetric PowerFill and Admira Fusion X-tra BCR (*p* < .05). Light intensity had no significant influence on the DoC for any of the BCR materials. The top layer of all four BCR materials resulted a significantly lower DoC compared to layer 3 and 4 regardless of the BCR materials (*p* < .05). A positive correlation was found between the hardness and DoC for all BCR materials (Figure 6).

**Table 5.** Degree of conversion (%) in all four RBCs cured by a monowave and a polywave light curing unit in various light intensities and corresponding curing times.

Light curing units	Curing time (seconds)	Layer	Filtek	Beautifill	PowerFill	Admira
Monowave	20s	Layer 1	30.62	47.66	75.23	58.35
		Layer 2	34.68	62.16	79.65	77.69
		Layer 3	45.32	63.80	80.56	84.48
		Layer 4	45.59	70.98	82.69	87.53
Polywave	3s	Layer 1	33.37	36.46	54.57	41.42
		Layer 2	33.37	41.16	62.02	42.09
		Layer 3	34.68	55.97	71.06	65.25
		Layer 4	57.28	63.05	72.83	68.47
	5s	Layer 1	41.96	34.46	57.13	53.57
		Layer 2	48.94	36.18	68.85	73.30
		Layer 3	48.94	67.03	89.21	84.98
		Layer 4	57.30	67.03	89.21	84.99
	10s	Layer 1	43.35	40.91	68.50	42.02
		Layer 2	45.32	40.69	86.97	51.57
		Layer 3	46.94	47.05	87.90	71.10
		Layer 4	48.94	67.03	89.21	74.99
	20s	Layer 1	43.06	36.18	64.06	68.53
		Layer 2	52.21	44.08	68.85	69.96
		Layer 3	56.03	52.76	72.48	72.33
		Layer 4	57.30	80.75	73.70	73.30

## 4. Discussion

The aim of this research was to investigate the effect of light curing intensity and wavelength spectrum on heat generation and chemomechanical properties of four brands of BCRs. Previous literature has suggested that there is a positive association where the mechanical strength is directly related to increased light curing intensity and that polywave LCUs results in uneven cure of the composite and reduction of overall mechanical properties [3,4, 8,9,11,12]. A similar relationship between the wavelength spectrum and mechanical properties was found in the present study, however, there was no correlation between the heat generation and the hardness.

Within the limits of this study, our null hypotheses were rejected. The LCU types and light intensity significantly affected the measured temperature increase and pattern of each composite (*p* < .05). There was a significant difference in temperature change when compared between monowave and polywave LCUs with different light intensity and corresponding curing time (*p* < .05). A positive correlation was found between hardness and degree of conversion in BCR materials. However, no correlations were found between the light curing intensity and hardness, nor between the temperature change and microhardness of the BCR materials.

The current study observed that the change in temperature was proportional to the increase in light intensity. This is consistent with the findings from previous literature [4,14]. Temperature increase during resin curing is a function of the rate of polymerization, as a result of exothermic polymerization reaction and the energy absorbed during polymerisation [15]. An increase in light intensity means an increase in light energy density. This can result in more energy being absorbed during polymerisation in the form of heat thus lead to an increase in temperature [16]. Previous studies reported that a temperature rise of 5.5–5.6 °C could cause some adverse effects on the pulp [17,18]. The critical temperature rise that causes pulp necrosis and the duration required is still controversial, however, it is evident that pulp temperature rise should be kept as low as possible during the polymerization of resin materials to avoid any risk of harming the pulp [17,18], emphasising the importance of the appropriate selection of LCUs and curing intensity.

Mechanical properties of BCRs are important influential factors that affect the longevity of the restoration. Hardness is an important determinant of mechanical property of BCRs as it reflects the depth of cure. In this study, BCR materials resulted in significantly higher hardness values with the application of monowave LCU compared with polywave LCU. This finding was consistent with previous literature and was thought to be associated with the limited transmission of violet wavelength as well as the inhomogeneity of irradiance of light beam [5]. The obtained hardness from the current study was in good agreement with El-Safty et al. [19] and Drummond [20], having the mean hardness range of 0.73 GPa to 1.60 GPa [19,20]. This can be associated with the use of photoinitiators in the BCR composites. In light-cured composites, photoinitiators are added to enable controlled polymerisation when photoactivated by a LCU emitting light at required wavelength. The most commonly used photoinitiator system in BRCs is camphorquinone (CQ), which has an absorption peak of approximately 470 nm that matches the wavelength emitted by most of the light-emitting diode (LED) LCUs on the market. A study conducted by Par et al. [4] concluded that the benefit of using a higher-irradiance multiple-peak curing unit was found only in composites containing alternative photoinitiators, such as Lucirin, which can be activated by a broad spectrum of light sources. However, as the shorter violet wavelengths have limited penetration compared to the blue light, there is a risk of insufficient and uneven polymerisation [10]. For thicker layer of BCRs, this is especially important because there is more composite for the light to travel through before reaching the bottom surface of the composite. This has further implications because an uneven or inadequate polymerization of the composite may lead to premature failure of the restoration due to problems such as increased wear and marginal breakdown [21].

No correlations were found between the light curing intensity and hardness of the BCR materials. This is thought to be associated with material composition, which is different in all four BCRs (Table 1). This includes the organic matrix, and the type and percentage of filler particles, which determine the light transmission [22,23]. Filler particles tend to scatter the light depending on filler particle size and content. Studies have shown that smaller filler particles with diameters approaching half the wavelength of light used for curing increase light scattering. Therefore, an increase in filler particle size tends to reduce scattering which in turn increases light transmission and the consequently the depth of cure [23–25]. Increasing in silica particle size is another factor associated with the reduction of the depth of cure and degree of conversion [23,26]. Higher filler content tends to reduce light transmission due to the increased probability of light refraction at the interfaces between the filler particles and the resin because of differences in their refractive indices [22,27]. Another factor that leads to lack of correlation found in this study is the different refractive indices among the four BCR materials. Past studies have shown that dental composites have a wide range of refractive indices depending on the particle size and its dispersion patterns, which can scatter the light and increase the hardness of the material [28,29]. This may be the reason why there is no positive correlation between the light curing intensity and the surfaces hardness of the four BCR studied; despite the short duration of light curing or lower light curing intensity, the light may be refracted between the particles, increasing the polymerisation of the overall BCR and hence the hardness. Therefore, further research is needed to assess the effect of different reflective indices on microhardness and light curing intensity.

Another important finding from this study is that a positive correlation was found between the hardness and degree of conversion in BCR materials, which is supported by a study conducted Ferracane [21]. This is associated with an adequate polymerisation reaction, affected by both the light source and the composition of the BCRs [24,30]. This positive correlation suggests the importance of sufficient polymerisation of BCR materials to promote maximal degree of conversion to ensure optimal mechanical properties of the BCR material, thus promoting successful long-term outcome and patient satisfaction.

A strength in this study conducted is the attempt to control confounding and prevent information bias through using tooth-coloured moulds to mimic the light reflection and refraction index of a tooth. The angulation of the light curing unit tip and distance to the surface layer of the BCR was also controlled through the use of clamp. However, the current study also has several limitations. This study was an *in vitro* study, meaning that the tooth mould used lacks of blood circulation and the consequent potential for heat dissipation leading to overestimation of temperature increase compared to vital human dentition. Future studies would be beneficial to incorporate the blood circulation and a 37 °C baseline-temperature set up to investigate in depth, the effect of material composition on light refractive index and subsequent light transmission. A further study looking into the possible influence of the dentine layer between the resin composite and the pulp, as well as the natural cooling of the pulp caused by the blood circulation would be beneficial.

## 5. Conclusion

Within the limitation of the current study, the following conclusions were drawn:

1. The change in temperature in BCR is proportional to the increase in light intensity.
2. BCR materials resulted in significantly higher hardness values when cured with monowave LCU compared with a polywave LCU.
3. No correlations were found between the light curing intensity and hardness of the BCR materials.
4. A positive correlation was found between the hardness and degree of conversion in BCR materials.

## Data Availability

Data will be available upon request.

## References

[1] Haenel T, Hausnerová B, Steinhaus J, et al. Effect of the irradiance distribution from light curing units on the local micro-hardness of the surface of dental resins. Dent Mater. 2015;31(2):93–104. 25483935 10.1016/j.dental.2014.11.003

[2] Randolph LD, Palin WM, Watts DC, et al. The effect of ultra-fast photopolymerisation of experimental composites on shrinkage stress, network formation and pulpal temperature rise. Dent Mater. 2014;30(11):1280–1289. 25261362 10.1016/j.dental.2014.09.001

[3] Alkhudhairy FI. The effect of curing intensity on mechanical properties of different bulk-fill composite resins. CCIDE. 2017;9:1–6. 10.2147/CCIDE.S130085 PMC5330190 28260947

[4] Par M, Repusic I, Skenderovic H, et al. The effects of extended curing time and radiant energy on microhardness and temperature rise of conventional and bulk-fill resin composites. Clin Oral Investig. 2019;23(10):3777–3877. 10.1007/s00784-019-02807-1 30693403

[5] Shimokawa CAK, Turbino ML, Giannini M, et al. Effect of light curing units on the polymerization of bulk fill resin-based composites. Dent Mater. 2018;34(8):1211–1221. 29801683 10.1016/j.dental.2018.05.002

[6] Alnazzawi A, Watts DC. Simultaneous determination of polymerization shrinkage, exotherm and thermal expansion coefficient for dental resin-composites. Dent Mater. 2012;28(12):1240–1249. 23018083 10.1016/j.dental.2012.09.004

[7] Kim RJ-Y, Son S-A, Hwang J-Y, et al. Comparison of photopolymerization temperature increases in internal and external positions of composite and tooth cavities in real time: Incremental fillings of microhybrid composite vs. bulk filling of bulk fill composite. J Dent. 2015;43(9):1093–1098. 26159386 10.1016/j.jdent.2015.07.003

[8] Miletic V, Santini A. Micro-Raman spectroscopic analysis of the degree of conversion of composite resins containing different initiators cured by polywave or monowave LED units. J Dent. 2012;40(2):106–113. 22094322 10.1016/j.jdent.2011.10.018

[9] Price RB. Light curing in dentistry. Dent Clin North Am. 2017;61(4):751–778. 28886767 10.1016/j.cden.2017.06.008

[10] Shortall AC, Price RB, MacKenzie L, et al. Guidelines for the selection, use, and maintenance of LED light-curing units - Part 1. Br Dent J. 2016;221(8):453–460. 27767163 10.1038/sj.bdj.2016.772

[11] Arikawa H, Kanie T, Fujii K, et al. Effect of inhomogeneity of light from light curing units on the surface hardness of composite resin. Dent Mater J. 2008;27(1):21–28. 18309608 10.4012/dmj.27.21

[12] Price RBT, Labrie D, Rueggeberg FA, et al. Correlation between the beam profile from a curing light and the microhardness of four resins. Dent Mater. 2014;30(12):1345–1357. 25460008 10.1016/j.dental.2014.10.001

[13] Zorzin J, Maier E, Harre S, et al. Bulk-fill resin composites: polymerization properties and extended light curing. Dent Mater. 2015;31(3):293–301. 25582061 10.1016/j.dental.2014.12.010

[14] Balestrino A, Veríssimo C, Tantbirojn D, et al. Heat generated during light-curing of restorative composites: Effect of curing light, exotherm, and experiment substrate. Am J Dent. 2016;29(4):234–2240. 29178754

[15] Armellin E, Bovesecchi G, Coppa P, et al. LED curing lights and temperature changes in different tooth sites. Biomed Res Int. 2016;2016:1894672. 27195282 10.1155/2016/1894672 PMC4852368

[16] Guiraldo RD, Consani S, Souza A. S D, et al. Influence of light energy density on heat generation during photoactivation of dental composites with different dentin and composite thickness. J Appl Oral Sci. 2009;17(4):289–293. 19668987 10.1590/S1678-77572009000400005 PMC4327644

[17] Dias M, Choi JJE, Uy CE, et al. Real-time pulp temperature change at different tooth sites during fabrication of temporary resin crowns. Heliyon. 2019;5(12):e02971. 31872130 10.1016/j.heliyon.2019.e02971 PMC6911866

[18] Tjan AH, Castelnuovo J, Shiotsu G, et al. Marginal fidelity of crowns fabricated from six proprietary provisional materials. J Prosthet Dent. 1997;77(5):482–485. 9151267 10.1016/s0022-3913(97)70140-9

[19] El-Safty S, Akhtar R, Silikas N, et al. Nanomechanical properties of dental resin-composites. Dent Mater. 2012;28(12):1292–1300. 23031485 10.1016/j.dental.2012.09.007

[20] Drummond JL. Nanoindentation of dental composites. J Biomed Mater Res B Appl Biomater. 2006;78(1):27–34. 16278844 10.1002/jbm.b.30442

[21] Ferracane JL, Mitchem JC, Condon JR, et al. Wear and marginal breakdown of composites with various degrees of cure. J Dent Res. 1997;76(8):1508–1516. 9240388 10.1177/00220345970760081401

[22] Bucuta S, Ilie N. Light transmittance and micro-mechanical properties of bulk fill vs. conventional resin based composites. Clin Oral Investig. 2014;18(8):1991–2000. 10.1007/s00784-013-1177-y 24414570

[23] Fronza BM, Ayres A, Pacheco RR, et al. Characterization of inorganic filler content, mechanical properties, and light transmission of bulk-fill resin composites. Oper Dent. 2017;42(4):445–455. 28402731 10.2341/16-024-L

[24] Abed YA, Sabry HA, Alrobeigy NA, et al. Degree of conversion and surface hardness of bulk-fill composite versus incremental-fill composite. Tanta Dental Journal. 2015;12(2):71–80.

[25] Malhotra N, Mala K. Light-curing considerations for resin-based composite materials: a review. Part II. Compend Contin Educ Dent. 2010;31(8):584–588. 20960988

[26] Fujita K, Ikemi T, Nishiyama N. Effects of particle size of silica filler on polymerization conversion in a light-curing resin composite. Dent Mater. 2011;27(11):1079–1085. 21824650 10.1016/j.dental.2011.07.010

[27] Leprince JG, Palin WM, Hadis MA, et al. Progress in dimethacrylate-based dental composite technology and curing efficiency. Dent Mater. 2013;29(2):139–156. 23199807 10.1016/j.dental.2012.11.005

[28] Ilie N, Keßler A, Durner J, et al. Influence of various irradiation processes on the mechanical properties and polymerisation kinetics of bulk-fill resin based composites. J Dent. 2013;41(8):695–702. 23707645 10.1016/j.jdent.2013.05.008

[29] Shortall AC, Palin WM, Burtscher P, et al. Refractive index mismatch and monomer reactivity influence composite curing depth. J Dent Res. 2008;87(1):84–88. 18096900 10.1177/154405910808700115

[30] Galvao MR, et al. Evaluation of degree of conversion and hardness of dental composites photo-activated with different light guide tips. Eur J Dent. 2013;7:86–93. 23407620 PMC3571515

